# Levels of extracellular ATP in growth zones of Arabidopsis primary roots are changed by altered expression of apyrase enzymes

**DOI:** 10.1080/15592324.2025.2555965

**Published:** 2025-09-17

**Authors:** Greg Clark, Diana Vanegas, Ashley Cannon, Miranda Jankovik, Ryan Huang, Katherine A. Brown, Eric McLamore, Stanley J. Roux

**Affiliations:** a Department of Molecular Biosciences, University of Texas at Austin, Austin, USA; b Department of Environmental Engineering and Earth Science, Clemson University, Clemson, USA; c The Oden Institute for Computational Engineering and Sciences, University of Texas at Austin, Austin, USA; d Cavendish Laboratory, University of Cambridge, Cambridge, UK

**Keywords:** AtAPY1, AtAPY2, ectoapyrase, extracellular matrix, microsensor, transgenic

## Abstract

In both animal and plant cells extracellular nucleotides act as hormone-like signals regulating many important physiological and developmental responses. In plants, many of these responses have been studied in roots. Here we used an enzyme-based microsensor to measure the concentrations of extracellular ATP (eATP) within 2 µm of epidermal cell surfaces in growth zones of primary roots of wild-type and transgenic Arabidopsis seedlings. The concentration of eATP outside of growing wild-type roots was in the nanomolar range and was highest in in the elongation zone. The concentrations of eATP in wild-type roots were altered in two kinds of mutants, those that were overexpressing *AtAPY1* or *AtAPY2*, which encode apyrases (NTPDases) that regulate root and root hair growth, and those that were suppressed in the expression of these two transcripts. Our results indicate that the [eATP] measured varies inversely with the level of expression of these apyrases. Structural modeling of these two apyrases predicts active site configurations capable of binding ATP. Taken together these results favor the hypothesis that AtAPY1 and AtAPY2 regulate eATP levels in primary roots.

## Introduction

Extracellular ATP (eATP) regulates a variety of physiological processes both in animals^1^ and plants.^2^^,^^3^ In animals, eATP can bind to and activate two different kinds of purinoceptors, P2X and P2Y.^4^ In plants, at least two protein kinase receptors have been identified,^5^ and ongoing research may lead to the discovery of still more receptors. An increase in cytoplasmic calcium levels is a common early step in signal transduction pathways induced by activating the purinoceptors in both animals^6^ and plants.^7‐9^

In plants, eATP plays a critical role in regulating growth and development. For example, root hairs and cotton fibers have a biphasic response to applied eATP or eATPγS, with lower levels promoting growth and higher levels inhibiting growth.^10^^,^^11^ Abundant evidence also indicates that eATP is a wound signal and participates in biotic stress responses in plants.^12^

As with all signaling pathways, it is important for cells to control the level of the signal. In both animal and plant cells, nucleotide-mediated signaling is controlled in part by the activity of ectoapyrase (ecto-NTPDase) enzymes that regulate the level of extracellular nucleotides by catalyzing the conversion of NTPs to NDPs and NMPs.^4^ Among the earliest of these enzymes to be well characterized in plants was an apyrase in potatoes that is targeted to the extracellular matrix (ECM) and functions to limit levels of eATP.^13^

In Arabidopsis there is a family of seven apyrase genes.^14^ Two of these apyrases, AtAPY1 and AtAPY2, favor ATP as their substrate,^15^ and reports have indicated they can function as ectoapyrases.^16^^,^^17^ However, other reports have suggested that these two apyrases are mainly localized in the Golgi,^18‐20^ and that AtAPY1, in contrast to the potato apyrase, favors UDP and GDP over ATP as a substrate.^21^^,^^22^ These studies, which utilized tagged versions of AtAPY1 and AtAPY2, found no evidence for the localization of either of these two apyrases in the plasma membrane or ECM. A genetic approach to resolving this controversy would be to test how the suppression or over expression of *AtAPY1* and *AtAPY2* affects the [eATP] in Arabidopsis tissue that secretes ATP as it grows. This approach would require sensitive methods to quantify the low µM levels of eATP,^23^ and various methods have been used to do this.

Early approaches to measuring eATP in plant cells were done using a luciferase-based detection system. Extracellular ATP was first visualized in *Medicago truncatula* seedlings using a bacterially expressed cellulose binding domain (CBD)-luciferase.^24^ Subsequently, expressing luciferase targeted to the ECM by the addition of a signal peptide to the *N*-terminus of the enzyme in wild-type Arabidopsis was used to examine eATP levels both in roots,^25^ and in guard cells.^26^ This method allowed evaluation of the relative [eATP] in the ECM of these tissues, but did not quantify the [eATP]. More recently, a genetically encoded FRET based fluorescent eATP sensor was used to show that wounding and salt treatment induced an increase in eATP levels in Arabidopsis seedlings.^27^ In Arabidopsis roots, a histochemical approach that used glucuronidase fused with five different eATP-inducible genes revealed that expression of these genes was highly responsive to ATP treatment in the meristem and elongation zones of Arabidopsis primary roots.^28^ A third strategy for measuring eATP has been to use a self-referencing electro-chemical biosensor based on the ATP-dependent reactions of immobilized glycerol kinase and glycerol-3-phosphate oxidase on the surface of a metal electrode. This approach was first tested on plant tissue in wounding experiments.^29^ Cannon et al.^30^ used this same biosensor system to document a gravity-directed release of ATP that plays a role in polarization of *Ceratopteris richardii* fern spores.

In this study we used the self-referencing electrode to assay the levels of ATP outside the primary roots of 4-d old Arabidopsis seedlings growing on agar plates. We confirmed that the method could detect nanomolar concentrations of eATP outside the apical zones of wild-type roots, and that these levels varied in different regions of these roots, with the highest levels being in the root cap and elongation zones, as had been previously reported.^24^^,^^25^ We then utilized loss- and gain-of-function mutants for *AtAPY1* and *AtAPY2* to quantitatively evaluate whether changing their level of expression altered the [eATP] in the major growth zones of primary roots. Our results clarify the role of these two apyrases in maintaining the [eATP] at low enough levels to sustain the growth of primary roots.

## Methods

### Plant material

Arabidopsis thaliana ecotype Wassilewskija (Ws) were used as wild-type control for this study. The transgenic mutants used in this study were R2-4A, APY1 OE and APY2 OE. The RNAi mutant, R2-4A, is null for *APY2* and has an estradiol-inducible RNAi construct which suppresses *APY1* expression. RNAi construction and generation of the R2-4A line were as previously described.^17^*Apyrase 1* and *2* overexpressing transgenic mutants, APY1 OE and APY2 OE, were previously characterized.^31^^,^^32^

Seeds were surface sterilized by soaking them in 70% (v/v) ethanol for 1 min, then 20% (v/v) bleach for 10 min, and then washing them 4–5 times in sterilized water. Sterilized seeds were stratified at 4 °C for 3−4 d and grown on vertical plates containing solid ½ Murashige Skoog (MS) medium with 0.5% (w/v) MES and 1.0% (w/v) agar raised to pH 5.7 with 1 M KOH. For experiments with R2-4A, both Ws wild-type seeds and R2-4A seeds were germinated and grown in the same growth media with a final concentration of 4 μM estradiol. A 40 mM stock of estradiol (Sigma) in DMSO was diluted 10,000-fold in ½ MS plates for a final treatment concentration of 4 μM estradiol and 0.01% (v/v) DMSO. Plates were wrapped with 3 M Micropore, placed vertically in a growth chamber and grown for 4 d at 23 °C under 16 h light–8 h dark cycle. For the root growth assay, images were taken after 4 d of growth with a Nikon Coolpix 990 digital camera and ImageJ was used to measure primary root length.

### Materials and reagents

ATP, Chloroplatinic acid 8% (w/v), lead acetate 30% (w/v), Nafion 117 solution, cerium (IV) oxide nanoparticles dispersion (mean particle size of 12 nm, 10% (w/v) in H_2_O), S-nitroso-*N*- acetyl-D L-penicillamine (SNAP), and MS media were purchased from Sigma-Aldrich (St. Louis, MO). Glycerol kinase (GK), and glycerol-3-phosphate oxidase (G3PO) were purchased from MP Biomedicals (Solon, OH). Glycerol, L-ascorbic acid was purchased from Fisher Scientific (Pittsburgh, PA). Laponite RD was obtained from Southern Clay Products Inc. (Austin, TX). Tetraethyl orthosilicate (TEOS), potassium nitrate (KNO3), o-phenylenediamine and hydrochloric acid (37% (w/v) HCl) were obtained from Acros Organics (New Jersey, NY). Single-layer graphene oxide (GO) (Thickness: 0.8 to 1.2 nm; purity 99%) was procured from ACS Material (Medford, MA). Potassium ferrocyanide trihydrate (K_3_Fe(CN)_6_) was purchased from EMD chemicals (Billerica, MA). PBS and TRIZMA buffers were procured from Mediatech, Inc. (Manassas, VA). Pt/Ir microelectrodes (PI20033.0A10, 1–2 mm tip diameter) were obtained from MicroProbes, Inc (Gaithersburg, MD).

### ATP electrode fabrication

All details for electrode preparation can be found in Vanegas et al.^29^^,^^33^ First, a nanocomposite of reduced graphene oxide and nanoplatinum was first deposited on the tip of a Pt/Ir microelectrode through a series of dip coating and sonoelectrodeposition. To inhibit transport of root exudate organic acids (ascorbic acid, etc.), a Nafion membrane was incorporated on top of the graphene/platinum layer. The stratified bi-enzyme composite (i.e. distinct GK and G3PO layers) was immobilized on the surface of the modified microelectrode using a layered encapsulation/entrapment approach with a laponite nanoshell as the separation barrier. Electrodes were stored in TRIZMA buffer at 4 °C until used.

### NO electrode fabrication

A composite of reduced graphene oxide (rGO), nanoceria (nCe), and nanoplatinum (nPt) was deposited on the tip of each Pt/Ir microelectrode based on the methods described by Chaturvedi et al.^34^^,^^35^ As described above, a Nafion membrane was included to facilitate charge exclusion of organic acids. After formation of the Nafion membrane, a size exclusion membrane was electrodeposited by polarizing the electrode at +900 mV in a solution of 5 mM o-phenylenediamine and 0.1 mM ascorbic acid for 120 min. All electrodes were stored in PBS at 4 °C until used.

### *H*_*2*_*O*_*2*_ *electrode fabrication*

The sandwich metal-nanocarbon structure developed by Vanegas et al.^29^ was used for preparing all *H*_*2*_*O*_*2*_ electrodes. Nanoplatinum was deposited using pulsed sonoelectrodeposition at 10 V and a pulsing frequency of 500 mHz. A layer of rGO was deposited, and then a second layer of nanoplatinum (nPt) was immediately formed to create a sandwich rGO-nPt-rGO hybrid material. A Nafion layer was deposited on the electrode prior to use; all electrodes were stored in PBS at 4 °C until used.

### Microelectrode calibration

All electrodes were calibrated in liquid ½ Murashige Skoog (MS) medium with 0.5% (w/v) MES raised to pH 5.7 with 1 M KOH at room temperature by recording DC potential oxidative current after addition of the target analyte prepared from stock solution. For calibration, ATP stock solutions (1 mM) were prepared in nanopure water by dissolving ATP salt and vortex mixing for one minute. NO stock solutions were prepared based on SNAP degradation.^36^ Briefly, decomposition to NO was initiated by a Cu(I) catalyst to produce a stock solution of 0.1 mM NO; stock solutions were stored in the dark when not in use and all calibrations were performed in the dark. H_2_O_2_ stock solutions were prepared by diluting electrograde peroxide solution to 0.1% (v/v) in nanopure water.

### Root profiling experiments

Flux measurements were conducted using a self-referencing (SR) microelectrode assembly composed of a vibration isolation table with faraday cage, camera/zoomscope, and supporting electronic equipment described in detail by McLamore and Porterfield.^37^ All flux measurements were taken by continuously recording differential oxidative current (Δi) during oscillation of the microelectrode from a position within 1μm of the root surface (called the near pole) to a position 20 μm away from the root surface (called the far pole). Fick's first law was then used to calculate eATP or NO flux as follows:

$$J=-D\frac{\Delta C}{\Delta X}=-D\frac{\left(\frac{i_{\mathrm{near}}-i_{far}}{m}\right)}{\Delta X}$$

where: *J* is the analyte flux (pmol cm^−2^ s^−1^), *D* is the molecular diffusion constant of ATP (2.6 × 10^−6^ cm^2^ s^−1^) or NO (3.3 × 10^−5^ cm^2^ s^−1^), Δ*C* is the differential analyte concentration, *ΔX* is the excursion distance of the oscillating electrode, *i*_*near*_ is the oxidative current measured at the near pole, *i*_*far*_ is the oxidative current measured at the far pole, and *m* is the linear slope of the sensors calibration curve (pA nM^−1^).

All flux measurements were repeated in triplicate and validated with abiotic experiments as described by Chaturvedi et al.^35^ and Vanegas et al.^33^ Distances from root tips were normalized for roots measured using methods described in McLamore et al.^38^

### Control experiments

To ensure oxidative current measured with the ATP microsensor was not due to endogenous H_2_O_2_ or NO produced by the roots, control studies were conducted using 4-d-old Ws wild-type roots. First, H_2_O_2_ or NO flux was recorded along the root surface to determine the endogenous levels present. Next, the ATP microsensor was used to measure oxidative current during stepwise injection of either ATP, H_2_O_2_ or NO (i.e., calibration).

### Bioinformatics and modeling

Bioinformatics and three-dimensional modeling studies were undertaken using protein sequences for AtAPY1 and AtAPY2 (GenBank accessions AAF66599.1 and Q9SPM5.1, respectively).^15^ Sequence alignments were produced used the Clustal Omega multiple sequence alignment program.^39^*N*-terminal regions of AtAPY1 and AtAPY2 were analyzed using the TOPCONS, a consensus prediction server for membrane protein topology and signal peptides.^40^ For both AtAPY1 and AtAPY2, the first *N*-terminal 42-residues as these residues were removed to eliminate false positives as these residues were predicted to contain a transit peptide and/or a membrane anchoring helix. These same 42 *N*-terminal residues were also removed for template-based modelling using the I-TASSER web server.^41^^,^^42^ Structure-based ligand-binding site predictions were undertaken using ATPbind^43^ and the COACH meta-server.^44^^,^^45^

### Propidium iodide root staining and microscopy

Propidium iodide stain was prepared using propidium iodide solution (Sigma) to make a 100 mM stock. Before imaging, seedlings were submerged in the solution for 45 s then washed three times to remove any residual stain. Seedlings were then placed on a slide in liquid MS media and covered with a cover glass and sealed with clear nail polish. Images were obtained using a Zeiss Axiovert Fluorescent Light Microscope with a Cy5 cube at 10× magnification.

## Results

### Roots suppressed in apyrase expression show higher [eATP] compared to wild-type roots

**Figure 1.**
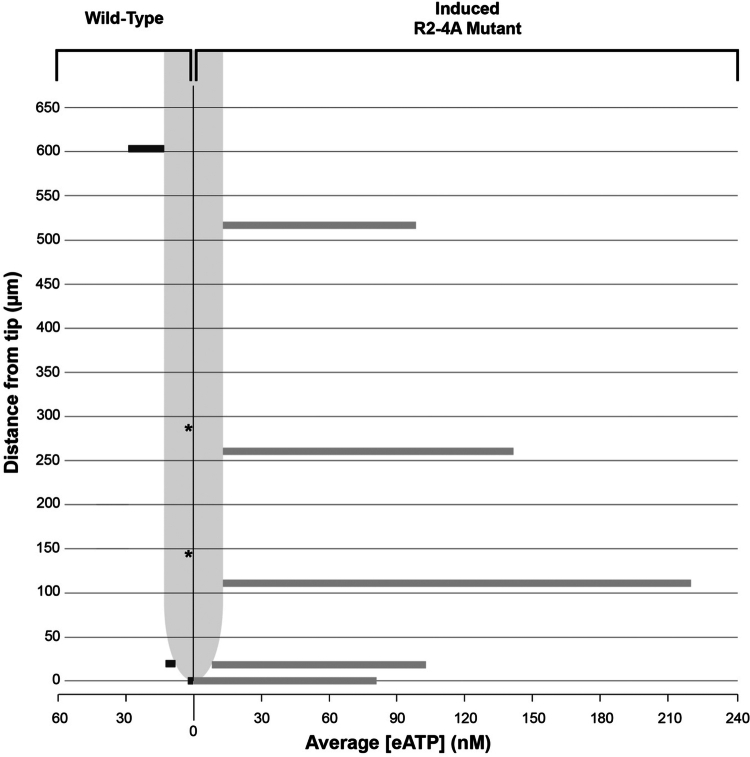
Representation of eATP concentrations outside an estradiol-treated primary root of 4-d old Arabidopsis seedlings at different distances from the tip of Ws wild-type and R2-4A apyrase loss-of-function plants. Levels of eATP outside of roots in which RNAi is induced to suppress expression of *AtAPY1* in the *atapy2* null mutant background (R2-4A) are higher at each location assayed compared to wild-type eATP levels. Average eATP concentrations determined for wild-type roots (*n* = 9) and for R2-4A roots (*n* = 7). Refer to Supplemental Table S1 for standard deviation values. *[eATP] at these root sites are < 4 nM.

Nanomolar concentrations of extracellular ATP (eATP) are detected outside of Ws wild-type and apyrase loss-of-function roots of 4-d old Arabidopsis seedlings. There are differences in eATP levels in different zones of the wild-type roots, with the highest concentrations in the root cap region and in the elongation zone (Figure 1, Supplemental Table S1). R2-4A seedlings are null for *AtAPY2* and, when grown in the presence of 4 μM estradiol, their expression of *AtAPY1* is suppressed by 70% after 2 d of growth.^16^ In estradiol-induced R2-4A seedlings, the concentration of eATP outside of primary roots ranges from 3- to 100-fold higher compared to that in Ws wild-type roots (Figure 1, Supplemental Table S1, Supplemental Figure S1). There are differences in the level of eATP along the R2-4A roots, with highest levels at the root tip in the root cap region and the elongation zone, which correlates with the regions of highest [eATP] found in Ws wild-type roots. Supplemental Figure S1 shows the microprofiles of [eATP] of Ws wild-type and R2-4A primary roots over the entire length of the root. Supplemental Figure S2 shows primary roots of Ws wild-type 4-d old seedlings stained with propidium iodide to confirm the location of the root cap, meristem, transition zone and elongation zone, based on description of these zones by Verbelen et al.^46^ Primary root growth was significantly inhibited in 4-d old seedlings grown in the presence of 4 μM estradiol as previously reported (Supplemental Figure S3).

### Roots overexpressing AtAPY1 or AtAPY2 have lower [eATP] than wild-type roots

The concentration of eATP outside of the primary roots of AtAPY1 OE and AtAPY2 OE seedlings ranged from 0.4 to 0.6 nM at each location outside of the root (Table 1; also see Supplemental Figures S4, S5). In contrast to Ws wild-type roots, there are also no observable differences in [eATP] in the different regions of the primary roots for AtAPY1 OE and AtAPY2 OE seedlings.

**Table 1.** Average concentration of eATP along the root surface of 4-d old Arabidopsis Ws wild-type and *AtAPY2* and *AtAPY2* overexpressing transgenic seedlings. Extracellular ATP (eATP) levels are greatly reduced outside of growing roots overexpressing either *AtAPY1* (APY1 OE) or *AtAPY2* (APY2 OE). Average eATP concentrations determined for Ws wild-type roots (*n* = 9) and for APY1 OE and APY2 OE roots (*n* = 4−8, SD = standard deviation. All error bars represent standard deviation of the arithmetic mean (*n* = 9 for wild-type, *n* = 4−8 for APY1 OE and APY2 OE; *α* = 0.05).

Wild-type				APY1 OE			APY2 OE	
Distance from root tip - μm	[eATP] nM	SD	Distance from root tip - μm	[eATP] nM	SD	Distance from root tip - μm	[eATP] nM	SD
0	2.3	0.35	0	0.48	0.18	0	0.44	0.20
19	12.2	1.5	20	0.31	0.18	20	0.56	0.21
143	2.2	1.4	140	0.45	0.19	130	0.49	0.26
287	3.3	2.5	307	0.50	0.29	332	0.51	0.23
603	28.8	0.38	603	0.44	0.32	606	0.56	0.17
2000	1.8	0.29	1503	0.41	0.28	1233	0.63	0.21

### *Microelectrode measurements of extracellular H*_*2*_ *O*_*2*_ *, NO and ATP indicate eATP measurements along primary roots are accurate*

In order to test the accuracy of flux microelectrode measurements we performed a control study and also measured the flux of H_2_O_2_ and NO to rule out any false positives with the eATP flux experiments. The flux of eATP was measured by utilizing an electrode developed by Vanegas et al.^33^ The self-referencing (SR) ATP microsensor developed by Vanegas et al.^33^ has been previously used to study eATP flux in germinating spores and growing *Zea mays* L. roots following touch stimulus^33^ and determine eATP flux/levels in germinating *Ceratopteris richardii* spores responding to gravity.^30^ The biosensor is based on the activity of two enzymes (glycerol kinase (GK) and glycerol-3-phosphate oxidase (G3PO)) organized in a stratified thin film that is segregated by a silica nanoshell; a graphene layer is used to enhance electrochemical signal transduction and improve sensitivity and response time. Chaturvedi et al.^35^ developed a NO-selective microelectrode based on a graphene-nanoceria hybrid composite and used the sensor in the SR modality to record NO flux from in three model organisms, including: bacteria, plant, and an invertebrate animal. Vanegas et al.^29^ developed a graphene-nanoplatinum electrode that has been used as a highly sensitive H_2_O_2_ electrode in biological studies.

When used in the SR modality (see McLamore and Porterfield),^37^ ATP, H_2_O_2_, or NO flux can be directly measured without addition of external reagents or contact with the plant tissue. Furthermore, the detection limit of each sensor is significantly lower than other published microelectrodes for studying plant roots.^29^^,^^33^^,^^34^

H_2_O_2_ is an endogenous radical that is electroactive and thus can possibly be measured as a false positive when using an oxidative potential of +700mV for detecting eATP. NO is also electroactive but cannot be measured below oxidative potentials of +720 mV and is less of a concern as a false positive for experiments herein. Nonetheless, control studies were conducted for both H_2_O_2_ and NO to determine if endogenous radicals were diffusing across the biomembrane on the tip of the electrode and causing false positive signals.

**Figure 2.**
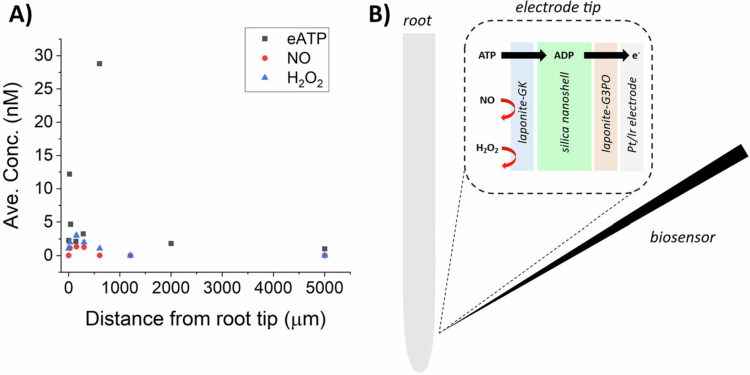
(A) Representative measurements of extracellular hydrogen peroxide concentrations ([H_2_O_2_]), nitric oxide concentrations ([NO]) and ATP along Ws wild-type roots measured with SR microelectrodes. (B) ATP interaction with glycerol kinase in the outermost layer leads to an enzymatic cascade that produces electrochemical signal. The laponite and silica protein nanoshells serve as barriers for NO or H_2_O_2_ diffusion. GK = glycerol kinase; G3PO = glycerol-3-phosphate-oxidase.

Using H_2_O_2_- or NO-specific microelectrodes,^29^^,^^35^ the concentration and flux of H_2_O_2_ and NO were recorded along the root surface to determine the endogenous levels near Ws wild-type roots (*n* = 5 roots). The maximum concentration of H_2_O_2_ and NO were recorded at a position that was approximately 150 μm from the root tip, normalized for root curvature (Figure 2A). The concentration of hydrogen and nitrogen radicals was exponentially lower in the shootward direction and had a value near 1 nM at the root tip. As shown by Vanegas et al.,^33^ the ATP biosensor is insensitive to H_2_O_2_ or NO due to a stratified bi-enzyme layer on the surface, which deters diffusion of these radicals from the liquid to the electrode surface (Figure 2B). Analytical selectivity of the ATP sensor over H_2_O_2_ or NO is shown in Supplemental Figure S6.

Because the average concentration of H_2_O_2_ 150 μm from the tip (3.1 ± 0.3 nM) was higher than the endogenous NO level (1.4 ± 0.2 nM) and both of these levels were similar to eATP levels measured for Ws wild-type roots (2.1 ± 1.4 nM), we tested whether these endogenous levels of H_2_O_2_ and NO lead to signal artifact for the ATP microsensor. Comparing the ATP biosensor output in the presence of endogenous radical levels and with the response to the calibration plot for ATP, the sensor specificity toward ATP is approximately 97% higher than the response produced by H_2_O_2_ or NO (Supplemental Table S2).

Although H_2_O_2_ and NO radicals are electroactive and can be measured with an oxidative potential of +700 mV, the control studies indicate that the signal error due to the presence of endogenous oxygen–nitrogen radicals is minimal and the reported ATP concentration and flux data is minimally affected by these radicals.

This selectivity for ATP over the radicals is due to the relatively thick enzyme membrane on the tip of the electrode. To produce a false positive signal, radicals are required to diffuse from the buffer into the outer enzyme membrane (glycerol kinase and laponite gel), across the orthosilicate nanoshell, through the second enzyme membrane (glycerol 3 phosphate oxidase and laponite gel), and finally across the Nafion membrane that coats the conductive nanomaterials (graphene and platinum). Diffusion of the radicals to the electrode surface is thus not energetically favorable, and we speculate that the radicals disassociate in solution (based on the short half-life of each compound). Although ATP is larger (i.e., higher diffusion coefficient) and also less electroactive than the radicals and is larger, the ATP is not required to diffuse across these four layers to produce an electrical signal. Rather, ATP interacts only with glycerol kinase in the outermost stratified layer on the electrode surface; a subsequent cascade of enzymatic reactions occurs within the membrane that produces electrical signal for the ATP biosensor.

### Bioinformatic analyses predict potential binding and catalysis of ATP

**Figure 3.**
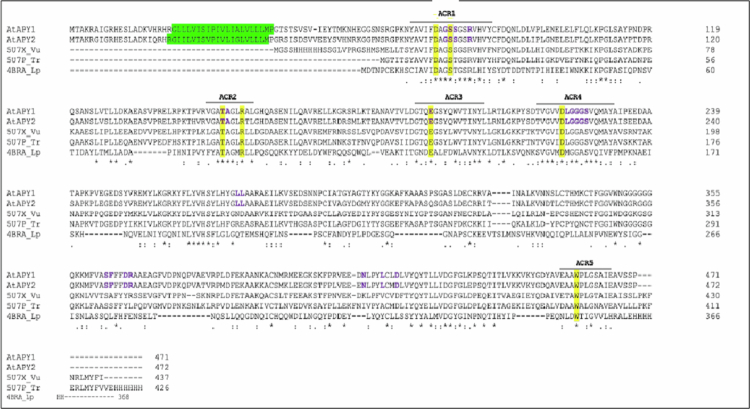
Alignment of AtAPY1 and AtAPY2 with sequences of related apyrases for which there is a crystal structure. Sequence alignment of residues of the full-length sequences for AtAPY1 and AtAPY2 are shown with sequences of residues in the primary structure of *V. unguiculata* (5U7X_Vu; Summers et al.^47^), *T. repens* (5U7P_Tp; Summers et al.^47^) and *L. pneumophila* (4BRA_Lp; Vivian et al.^49^). The predicted single-pass outward-facing transmembrane helix in the AtAPY1 and AtAPY2 sequences is highlighted in green. ACR sequence motifs are indicated by the bar beneath the 5 ACR labels, and the seven highly conserved residues required for diphosphohydrolase activity are highlighted in yellow.

Based on our microelectrode data indicating that the expression of *AtAPY1* and *AtAPY2* can control the [eATP] outside of roots, we performed bioinformatic analyses to assess the potential of the AtAPY1 and AtAPY2 enzymes to bind ATP. Figure 3 shows alignments of protein sequences of apyrases AtAPY1 and AtAPY2 with three structurally related NTPDases (*Vigna unguiculata* and *Trifolium repens*^47^^,^^48^ and *Legionella pneumophilia,*^49^ for which crystal structures exist. TOPCONS prediction^40^ of AtAPY1 indicated that its *N*-terminal sequence is likely to be a single-pass transmembrane helix,^50^ with an inside-to-outside orientation contained within the *N*-terminal 42 residues. A similar result was obtained for AtAPY2 (annotated in Figure 3). Both of these proteins could therefore be anchored in membranes with a globular C-terminal ecto-domain.

Template-based homology models for the predicted globular domains of AtAPY1 and AtAPY2 were generated use the I-TASSER webserver.^41^^,^^42^ The three-dimensional folds of both these models are most similar to the crystal structure of the legume *Vigna unguiculata* NTPDase (RCSB PDB entry 5u7x).^47^ An RMSD of 0.26 Å was reported for structurally aligned residues between 5u7X with either the AtAPY1 or AtAPY2 model. Both models share the same two-domain structure characteristic of apyrases. Apyrase folds are formed from two modified RNAase-H motifs assembled from alpha-helical and beta-sheet subdomains.^47^ This fold belongs to the Nucleotide Binding-Domain (NBD) of the sugar kinase/HSP70/actin superfamily.^51^ In this superfamily, substrate binding is associated with the conformational changes that close the active site cleft, and cleft closure can be modulated by allosteric effectors.^52^

**Figure 4.**
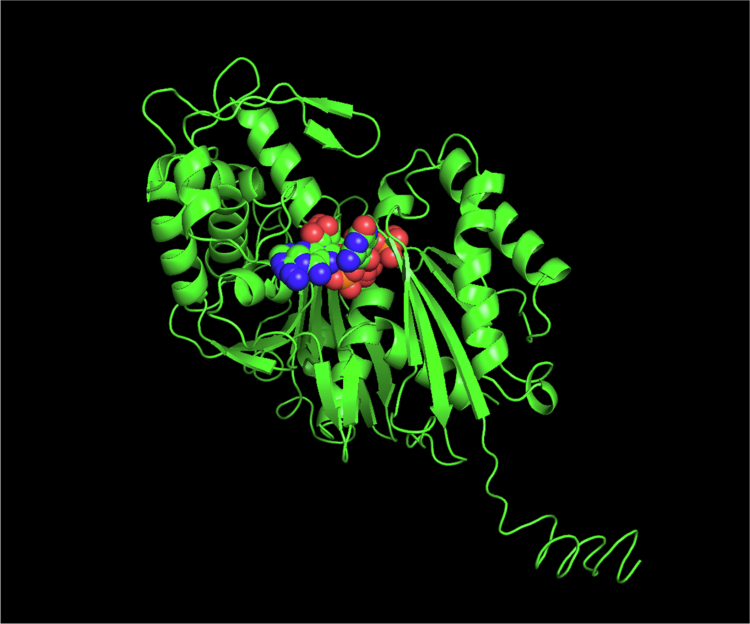
Model of AtAPY1 in complex with an ATP analog. The predicted fold of AtAPY1 is displayed as green ribbon. AMP-PNP, a non-hydrolyzable analog of ATP, is shown as spheres (image produced using the PyMOL Molecular Graphics System, Version 3.1, Schrödinger, LLC).

The predicted active sites of both AtAPY1 and AtAPY2 contain a number of features consistent with substrate binding and catalysis typical of NTPDases. These include apyrase conserved regions (ACRs) – five conserved protein motifs associated with the functional properties of the enzymes (annotated in Figure 3; Zebisch and Strater, 1996).^51^^,^^53^ Seven residues within these ACRs have been identified to be mechanistically important in the diphosphohydrolase reaction of NTPDases and are present in the active sites of the *V. unguiculata* and *T. repens* legume NTPDase crystal structures.^47^ These residues are also conserved in the AtAPY1 and AtAPY2 protein sequences (Figure 3). Their homology models share a similar spatial arrangement with the same residues in the *V. unguiculata* and *T. repens* legume NTPDase crystal structures.^47^ Analysis of the homology models using the ATPbind meta-server^43^ predicted the presence of an ATP binding site in both AtAPY1 and AtAPY2. Additionally, the COACH meta-server^44^ identified ligand-binding sites in both AtAPY models for nonhydrolyzable analogues of ATP (AMP-PNP; 5′-*O*-(hydroxy{[hydroxy(phosphonoamino)phosphoryl]oxy}phosphoryl)adenosine) and ADP (annotated in Figure 4).

A representative modelled complex of AtAPY1 with AMP-PNP is shown in Figure 4. These binding sites share structure-based similarity with the active sites in the crystal structures of NTPDases from *L. pneumophilia* and *Toxoplasma gondii*.^49^^,^^54^^,^^55^ Clustering results also indicated some shared structural similarities with binding sites for AMP and nonhydrolyzable analogues of ADP, GTP and UTP observed in crystal structures of complexes with *L. pneumophilia*, *T. gondii* and rat NTPDases.^55^^,^^56^ Putative ligand-binding sites for Mg^2+^, used for nucleotide co-ordination, were also identified in the active sites of AtAPY1 and AtAPY2.

## Discussion

Many different root growth responses to eATP have been documented in seedling roots, including the elongation of primary roots and root hairs, lateral root formation and growth, root skewing, and root avoidance.^11^^,^^17^^,^^20^^,^^32^^,^^57^^,^^58^ eATP also induces multiple physiological responses in primary roots, including a rapid depolarization of root hair cells,^59^ changes in auxin transport and accumulation,^60^^,^^61^ and the induction of Ca^2+^ waves that are modified by phosphate nutrition status.^62‐64^ Using a microelectrode approach we directly measured nanomolar [eATP] along primary root tips and determined that [eATP] varies in different tip regions. In order to better understand the role of apyrases in eATP signaling during plant root responses, we quantified how different levels of apyrase expression affected eATP concentrations in different zones of growing roots of wild-type Arabidopsis seedlings.

A major way ATP is transported into the ECM is by its release from ATP-containing secretory vesicles that cells deliver to the ECM during their growth.^17^^,^^65^ High [eATP] can inhibit auxin transport and growth,^16^^,^^60^^,^^61^ but the increased apyrase activity observed in the ECM of growing cells helps to keep the [eATP] below inhibitory levels.^16^^,^^17^ Correspondingly, the inhibition of ectoapyrase activity allows the concentration of ATP released into the ECM during growth to increase to levels that inhibit auxin transport,^60^ which results in the inhibition of growth. However, the dual inhibition of apyrase expression and auxin transport also increases the delivery to the ECM of secretory vesicles containing wall peroxidases that promote cross linking and decrease wall extensibility,^16^ and the delivery of these vesicles would further increase [eATP].

The nanomolar ATP concentrations (1−30 nM) outside the epidermal cells of WT roots are in the range that could initiate signaling responses, based on the dissociation constant [Kd] of the two ATP receptors; i.e., 45.7 ± 3.1 nM for P2K1 and 44.7 ± 15.73 nM for P2K2.^5^^,^^66^ The observation of differences in the eATP level depending on the tissue-type, with higher levels of eATP in the root cap and elongation zone of the root, is consistent with the hypothesis that more ATP is released from cells that are actively secreting or expanding.^65^ The highest concentration of eATP was found near 600 µm from the root tip, which is in the rapid elongation zone, containing cells with the fastest growth rate in the root tip.^46^ Notably, an eATP treatment was found to induce a transient depolarization of plasma membrane voltage and an increase in [Ca^2+^]_cyt_ in epidermal cells in the elongation zone of Arabidopsis seedlings.^7^ Our results generally correlate with the findings of Kim et al.^24^ but provide more detailed information about the eATP concentrations outside different growth zones of seedling primary roots.

Currently the question of whether Arabidopsis AtAPY1 and AtAPY2 are capable of using ATP as a substrate is controversial. The original biochemical studies of these two Arabidopsis apyrases found that after they were expressed in bacteria and highly purified, they showed highest NTPDase activity using ATP as the substrate,^15^ and a more recent dissertation report found that untagged AtAPY1 that was highly purified from the nuclei of etiolated Arabidopsis seedlings favored ATP as its substrate.^67^ Another study documented a two-fold increase in the level of eATP in samples from the media of liquid-grown R2-4A *AtAPY1*/*AtAPY2* loss-of-function seedlings compared to samples from the media of liquid-grown wild-type seedlings.^16^ Consistent with these reports, the modelling studies presented here suggest that both enzymes appear to have residues distributed within a binding pocket that would predict their ability to bind ATP as well as AMP, ADP, GTP and UTP based on comparisons with existing crystal structure complexes. Other studies, however, found that a GFP-tagged version of AtAPY1 isolated from light-grown Arabidopsis, or a His-tagged version of AtAPY1 isolated from HEK293 cells had little or no activity toward ATP substrates, but, instead, favored GDP and UDP substrates, indicative of a Golgi-related apyrase function.^68^

Even minor modifications of apyrases can alter their substrate preference.^4^ Thus, the addition of GFP or His tags to AtAPY1, or the post-translationally modified state of AtAPY1, depending on the source cell, tissue, or organelle from which it is isolated, could bias which nucleotide it favored as its substrate. More studies relating the structural state of apyrases to which substrate nucleotides they favor are needed, but the different structural states of the AtAPY1 tested in prior reports could help explain the contrasting conclusions in these reports about its role in regulating the [eATP].

## Conclusions

Microelectrode measurements of eATP along the outside of the primary roots of Arabidopsis seedlings directly quantified nanomolar concentrations of eATP that are consistent with the well-documented role of eATP as a hormone-like signal in plants. Different [eATP] were found in different regions of wild-type roots with the highest levels in the root cap and elongation zone. Compared to wild-type roots, there were large increases in eATP levels in all regions of R2-4A loss-of-function roots; in contrast, constitutive overexpression of *AtAPY1* or *AtAPY2* resulted in much lower eATP levels all along the root. The new data provided in this study supports the conclusion that AtAPY1 and AtAPY2 function to limit root eATP levels in Arabidopsis seedlings. These *in vivo* results show an inverse correlation of the expression of AtAPY1 and AtAPY2 with the [eATP] in primary roots. Together with new structural evidence that these enzymes are capable of binding ATP to use as a substrate, they favor the hypothesis that these apyrases can play a major role in controlling the [eATP] in Arabidopsis seedings.

## Supplementary Material

Supplementary material Supplementary Tables.

Supplementary material Supplementary Figures.
