# Supplementary material for: Levels of extracellular ATP in growth zones of Arabidopsis primary roots are changed by altered expression of apyrase enzymes
Source: Plant Signal Behav. 2025 Sep 17;20(1):2555965. doi: 10.1080/15592324.2025.2555965 (PMC12445451; doi:10.1080/15592324.2025.2555965)
Supplement: Supplementary material — Supplementary Tables. [file KPSB_A_2555965_SM7154.docx]

**Supplemental Table S1.** Concentration of eATP along the root surface of estradiol-treated Arabidopsis Ws wild-type and R2-4A transgenic apyrase loss-of-function 4-day old seedlings. In wild-type seedlings, the highest average [eATP] is observed in the elongation zone and root cap. Levels of eATP outside of roots in which RNAi is induced to suppress the expression of *AtAPY1* in the *atapy2* null mutant background (R2-4A) are higher at each location assayed compared to wild-type eATP levels. Average eATP concentrations determined for wild-type roots (n=9) and for R2-4A roots (n=7), SD = standard deviation of the arithmetic mean (α= 0.05).

| **Wild-type** | | | **R2-4A** | | |
| --- | --- | --- | --- | --- | --- |
| **Distance from root tip - μm** | **[eATP] nM** | **SD** | **Distance from root tip - μm** | **[eATP] nM** | **SD** |
| **0** | **2.3** | **0.35** | **0** | **81** | **23.9** |
| **19** | **12.2** | **1.5** | **18** | **112.6** | **78.2** |
| **143** | **2.2** | **1.4** | **111** | **220.9** | **39.1** |
| **287** | **3.3** | **2.53** | **260** | **141.9** | **65.1** |
| **603** | **28.8** | **0.38** | **519** | **98.6** | **17.9** |
| **2000** | **1.8** | **0.29** | **1210** | **35.8** | **29.1** |

**Supplemental Table S2.** Key performance indicators of ATP microelectrode and selectivity data based on the data in Supplemental Figure S6. The microsensor had a LOD toward eATP of 1.1 nM and was more than 4 times selective over hydrogen peroxide and more than 12 times selective over nitric oxide.

| **Analyte tested** | **Analytical Sensitivity [uA/nM]** | **R^2** | **LOD** | **Range [nM]** | **Selectivity for ATP [%]** |
| --- | --- | --- | --- | --- | --- |
| ATP | 14.4 | 0.98 | 1.1 | 1.1 to 5.0 | - |
| H_2_O_2_ | 3.7 | 0.04 | N/A | N/A | >4 times |
| NO | 1.2 | 0.01 | N/A | N/A | >12 times |
