# Supplementary material for: Levels of extracellular ATP in growth zones of Arabidopsis primary roots are changed by altered expression of apyrase enzymes
Source: Plant Signal Behav. 2025 Sep 17;20(1):2555965. doi: 10.1080/15592324.2025.2555965 (PMC12445451; doi:10.1080/15592324.2025.2555965)
Supplement: Supplementary material — Supplementary Figures. [file KPSB_A_2555965_SM7153.docx]

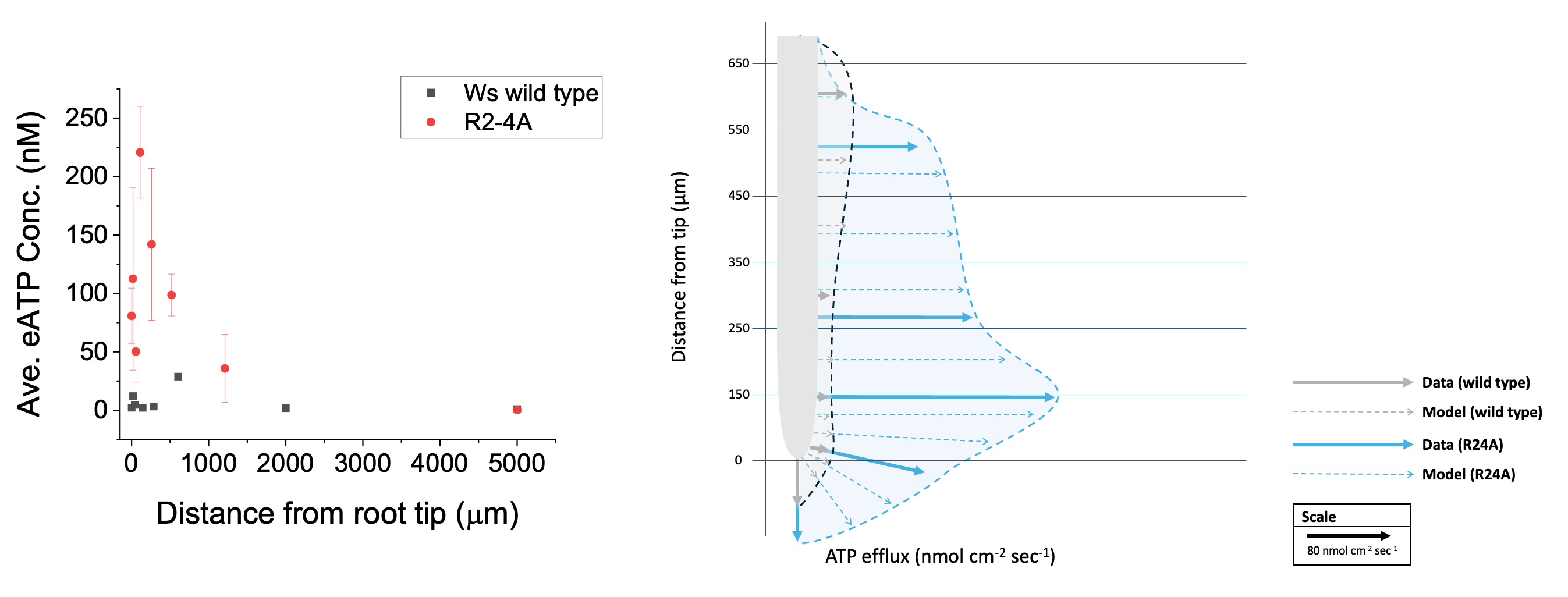


**Supplemental Figure S1.** ATP efflux profiles near wild type (grey) and R2-4A roots (blue) show distinct physiological patterns. **A)** Microprofile for average nanomolar concentrations of eATP at different distances (µm) from the root tip of primary roots of estradiol-treated Ws wild-type and R2-4A 4-day old seedlings. **B)** ATP microprofile data are indicated with solid arrows and model data are indicated with dashed arrows/lines. The legend shows scale bar for interpreting values of ATP flux (in units of nmol cm^-2^ sec^-1^)

**
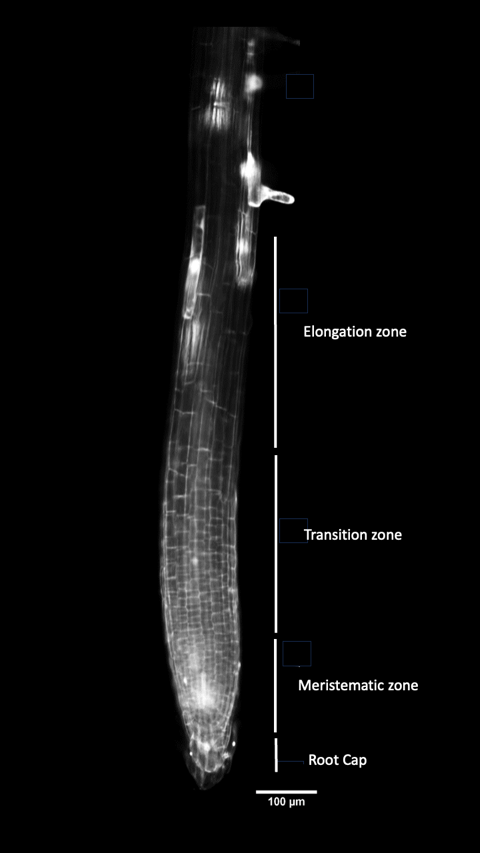
**

**Supplemental Figure S2.** Propidium iodide staining of primary roots of 4-day old Ws wild-type Arabidopsis seedlings.

**
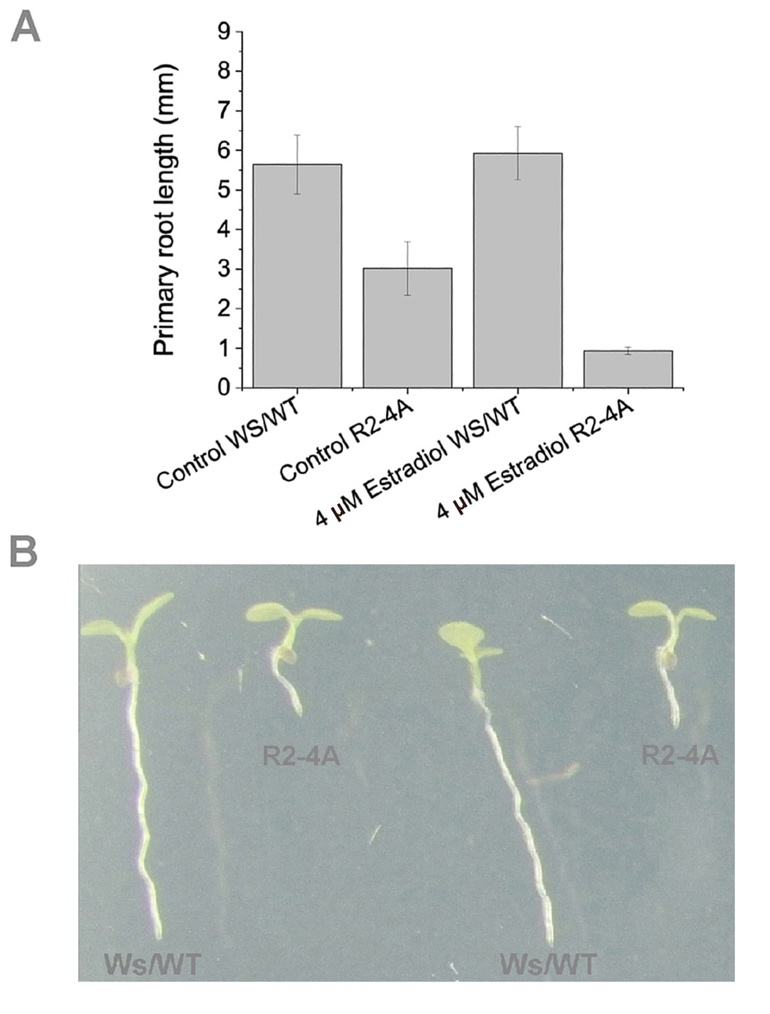
**

**Supplemental Figure S3. A)** Primary root lengths of control and estradiol-treated 4-day old Arabidopsis Ws wild-type (WS/WT) and R2-4A seedlings. Error bars represent standard deviation. Different letters above the bars indicate mean values that are significantly different from one another (p < 0.05). **B)** Representative images of 4-day old Arabidopsis Ws wild-type (WS/WT) and R2-4A seedlings treated with 4 μM estradiol.

**Supplemental Figure S4.** Microprofile for average nanomolar concentrations of eATP at different distances (µm) from the root tip of primary roots of APY1 OE and APY2 OE 4-day old seedlings. Error bars represent standard deviation (n=4-8; α= 0.05).


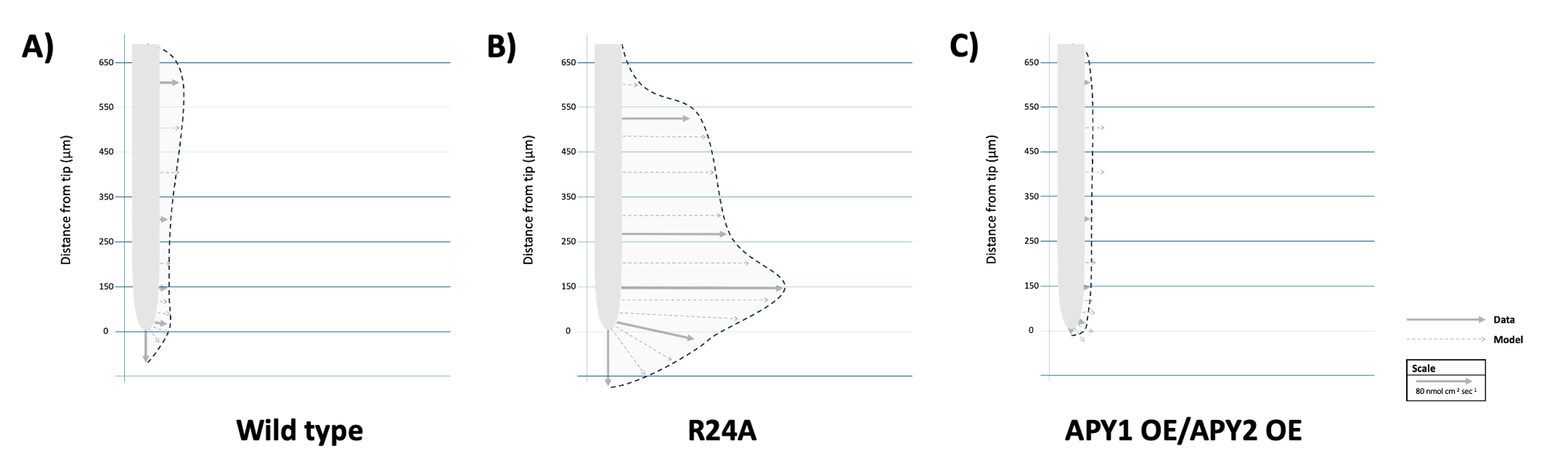


**Supplemental Figure S5.** The profiles of ATP concentrations near roots demonstrate distinct physiological patterns. **A)** Ws wild type. **B)** R2-4A. **C)** APY1 OE/APY2 OE. ATP microprofile data are indicated with solid arrows and model data are indicated with dashed arrows/lines. The legend shows the scale bar for interpreting values of ATP flux (in units of nmol cm^-2^ sec^-1^).


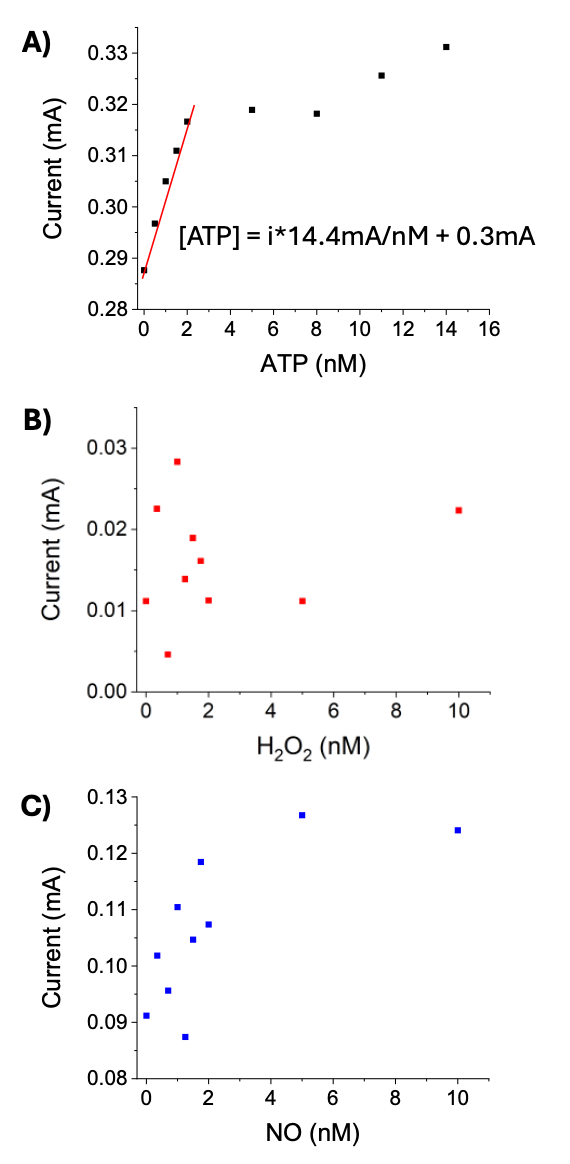


**Supplemental Figure S6.** Single-target calibration plot of microsensor for eATP and two potential interferent oxidants. This experiment was conducted to determine whether oxygen or nitrogen radicals (NO and H_2_O_2_) near the tissue surface would impact the eATP microsensor data. **A)** eATP calibration with a slope of 14.42mA/nM, **B)** eATP probe challenged with H_2_O_2_ in a selectivity test with a slope of 0.02µA/nM, and **C)** eATP probe challenged with NO in a selectivity test with a slope of 0.15µA/nM.
